# Molecular Markers in the Diagnosis and Treatment of Cancer

**DOI:** 10.1155/2015/105217

**Published:** 2015-09-14

**Authors:** Murat Gokden, Aurelio Ariza, Konstantinos Arnaoutakis

**Affiliations:** ^1^Department of Pathology, University of Arkansas for Medical Sciences, Little Rock, AR 72205, USA; ^2^Department of Pathology, Autonomous University of Barcelona, 08916 Barcelona, Spain; ^3^Hematology-Oncology Division, Department of Internal Medicine, University of Arkansas for Medical Sciences, Little Rock, AR 72205, USA

Our understanding of cancer as a disease process has evolved tremendously over the centuries, culminating in the late 20th century with the discovery of oncogenes and tumor suppressor genes and subsequent understanding of carcinogenesis as it is depicted in the classic hallmarks of cancer paper by Hanahan and Weinberg [1]. Genetic and epigenetic alterations have been increasingly identified in many diseases, including a wide variety of neoplasms. As more of these alterations are being discovered, their significance in some diseases remains still obscure, while they have become diagnostic, prognostic, and predictive genetic signatures for others.

It is becoming clear that a given genetic alteration and associated molecular changes involving particular pathways in the neoplastic cell may not necessarily be specific for that particular type of cancer. Rather, such a genetic alteration represents a more general abnormality involved in the neoplastic transformation of a variety of cancers in different organs. For instance, mutations in* BRAF* can be seen in unrelated cancers such as melanoma, colorectal and lung carcinomas [2, 3], brain tumors [4], and hematolymphoid malignancies [5]. This paves the way to potentially identifying which of these alterations a cancer has, rather than the classical diagnostic approach of which organ it originates from or what the histologic type is, essentially redesigning the cancer taxonomy. This disease or organ-agnostic type of approach is also the mainstay of a “personalized” approach to cancer treatment.

Some of these alterations are also used as diagnostic aids in differential diagnostic settings, such as IDH-1 R132H identification by immunohistochemistry or the identification of other* IDH-1 *or* IDH-2* mutations in diffuse gliomas, in contrast to well-circumscribed gliomas or reactive gliosis [6].

An increasingly growing number of these alterations are now the subject of targeted therapies especially in the form of small molecule kinase inhibitors. They can also provide significant prognostic (such as FLT-3 mutation in acute myelogenous leukemia) and predictive information, further blurring the boundaries between diagnosis and treatment, as well as between basic and clinical sciences. It is not enough anymore for pathologists to provide only diagnosis but also an array of molecular markers that facilitate the discussion about prognosis for given cancer and potential therapeutic options.

Of paramount importance are the explosion of knowledge in molecular biology and its clinical application in the form of molecular diagnostics, involving high-technology testing. Altogether, we have a better understanding of how such alterations operate in the process of oncogenesis, which in turn helps us better diagnose and treat neoplasms based on these alterations.

These discoveries have also influenced the pharmaceutical and biotechnological fields, resulting in development of additional treatment options for cancer patients: O^6^-methylguanine DNA methyltransferase (*MGMT*) gene methylation status in glioblastoma and response to alkylating agents [7],* KIT* mutations in gastrointestinal stromal tumor (GIST) and response to imatinib [8],* ALK* gene rearrangements in* ALK*-positive nonsmall cell lung carcinoma and response to crizotinib [9], and EGFR mutations in nonsmall cell lung carcinoma and response to gefitinib [10] are a few examples of how genetic alterations, identified by molecular diagnostic testing, can impact treatment decisions.

Despite this enormous success over the last 50 years since the discovery of DNA double helix and the discovery of the first human oncogene, there are still a lot of questions in regard to the optimal way of molecular testing, distinguishing between passenger and driver mutations in a tumor, dealing with the vast intra- and intertumor heterogeneity, and introducing other nongenetic molecular markers such as proteins (proteomics) and metabolites (metabolomics).

In this* special issue,* we present a variety of manuscripts that report technical, basic, and clinical research, molecular biology, and diagnostic and therapeutic aspects of neoplasia, as well as reviews of these subjects. The topics are not limited to a particular organ, system, or type of neoplasia. The manuscripts emphasize the importance of molecular markers in various aspects of neoplasia in an attempt to provide the reader with an up-to-date source of current research on molecular markers in cancer.
